# Vaccines, emerging viruses, and how to avoid disaster

**DOI:** 10.1186/s12915-014-0100-6

**Published:** 2014-11-29

**Authors:** Rino Rappuoli

**Affiliations:** Novartis Vaccines, Siena, 53100 Italy

## Abstract

Rino Rappuoli is a graduate of Siena University, where he also earned his PhD before moving to the Sclavo Research Center, the Italian vaccine institute, also in Siena. He then spent two years in the USA, mostly at Harvard with John Murphy and Alwin Pappenheimer working on a new diphtheria vaccine based on a non-toxic mutant of diphtheria toxin which has since become the basis for conjugate vaccines against *haemophilus*, *meningococcus*, and pneumococcal infections, before returning to the Sclavo Research Center where he developed an acellular vaccine based on a mutant pertussis toxin. With many achievements in vaccine development to his credit, he is now Global Head of Vaccines Research and Development for Novartis Vaccines in Siena, and has most recently pioneered reverse vaccinology, in which the genome of the pathogen is screened for candidate antigenic and immunogenic vaccine components. We spoke to him about the potential for outbreaks of the kind we are now seeing with Ebolavirus in West Africa, and what can be done to prevent them.

## Ebolavirus disease has been much in the news recently because of the horrendous outbreak in West Africa – but how many other rare, sporadic but severe infections are there that pose an equal threat?

Well, during the last few years, more or less with every 6 months to a year there has been a new outbreak of an emerging infection. Examples are the influenza viruses - for instance H1N1 in 2009, and H7N9 and H5N1 more recently [1] - the MERS rotavirus in Saudi Arabia [2], and Chikungunya virus in St Martin in 2013 [3], and of course now Ebola virus [4]. So there are many rare but potentially very dangerous diseases or emerging infections regularly breaking out. Ebola virus is by far the most lethal that we have now, but all of the others are very dangerous.

## I know that there are ’flu vaccines, but are there licensed vaccines for any of the others?

The answer is no - there are no vaccines for SARS, which emerged in 2003, there is no licensed vaccine for MERS, there is no licensed vaccine for Chikungunya, there is no licensed vaccine for Ebola - so for most of these diseases there are no vaccines, because they are too rare to justify the economic investment.

## Is that the only difficultly, or are there other difficulties in developing vaccines for these sorts of diseases? I realise it may be impossible to generalise.

Most of the time we do have the technologies to make these vaccines. I remember for example when SARS came in 2003/2004 we made a vaccine pretty quickly and brought it to the stage where it was ready to go to phase 1, but when we got to phase 1 the disease had gone away, there was no interest, no incentive to bring it even to phase 1, so we dropped it. For Ebola I think that the technology to make a vaccine has been there for many years, but nobody wanted to invest in it because there was no market, no demand.

## Do you think this is going to have to change in the light of what has happened with Ebola?

I hope that this humanitarian and health disaster is going to help us to become a little bit smarter in planning the future. I see these emerging infectious diseases as a constant threat. They pose a risk, just as in other areas we have a risk of earthquakes, we have a risk when we drive a car of car accidents, when we buy a house there is a risk of burning the house, and so on - and for all of those risks we prepare ahead of time. We have insurance for cars or we have insurance for houses and we build buildings that are earthquake-resistant. It looks as though the only place where we are not able to make investments ahead of time is health - we always have to wait for the disaster to happen and then we rush and usually with the vaccine we come too late and when the disease is no longer there then everyone forgets and we go from one disaster to another.

## How forward-thinking do we need to be? - How long does it actually take to develop a vaccine, generally?

For a normal commercial vaccine the time is between 10 and 15 years. Under very accelerated circumstances, like those we have now for Ebola, you can take advantage of previous studies that have been done and try to rush the development of the vaccine, but we do take risks with safety and efficacy when we accelerate so much, so I think we will be wise to develop those vaccines ahead of time.

## How much of a problem is viral mutation?

It depends on the virus, so for Ebola it is not a problem at all, for MERS it does not seem to be a problem, for SARS it does not seem to be a problem, for Chikungunya it does not seem to be a problem. For influenza it is a problem and for HIV it is a big problem, so it depends on the virus.

## What about the possibility, aired in the Press, that Ebola might become transmissible in an airborne form? Do you have any view on that possibility?

I believe that it is extremely unlikely. This virus has been there for a long time in the wild in Africa and it has never done it. It has had many opportunities to do it. I don’t think that a small exposure to mankind right now is going to do it and the preliminary data from the genetics and genomics of the virus show very good stability so I don’t think this is a risk in the short term.

## So a little bit of good news. But you say one of these diseases pops out about every six months. Do you expect that we’ll be continuing to see emerging infections we haven’t seen before?

Oh yes, I believe that in six months we will have a new emerging disease. Hopefully the Ebola outbreak will be under control. It will not be in the news and there will be other news about another emerging infection and we will rush into the new one, and instead of doing that I think it would be nice to be able to plan ahead of time. I think we have technologies today by which we could prepare multiple vaccine platforms that could be tested for safety and efficacy against multiple diseases and where you could plug in an antigen from a new emerging infection quickly. We already effectively have this for influenza virus, for which as the virus mutates each year we just plug in the most prevalent haemagglutinin variants to the existing vaccine platform. If the regulatory authorities could approve platforms suitable for use against other pathogens – thus already of proven safety and efficacy – we could have a way of being as ready as possible for unexpected events.
